# Haploinsufficiency of *NR3C1* drives glucocorticoid resistance in adult acute lymphoblastic leukemia cells by down-regulating the mitochondrial apoptosis axis, and is sensitive to Bcl-2 blockage

**DOI:** 10.1186/s12935-019-0940-9

**Published:** 2019-08-23

**Authors:** Haowen Xiao, Yingying Ding, Yang Gao, Li-Mengmeng Wang, Huafang Wang, Lijuan Ding, Xiaoqing Li, Xiaohong Yu, He Huang

**Affiliations:** 1 Department of Hematology, Sir Run Run Shaw Hospital, Zhejiang University School of Medicine, No. 3 Qingchun East Rd., Hangzhou, 310016 Zhejiang People’s Republic of China; 2 Institute of Hematology, Zhejiang University, Hangzhou, Zhejiang People’s Republic of China; 3 Department of Hematology, The People’s Hospital of Zhongshan City, Zhongshan, Guangdong Province People’s Republic of China; 4 Bone Marrow Transplantation Center, The First Affiliated Hospital, Zhejiang University School of Medicine, No. 79 Qingchun Rd., Hangzhou, 310003 Zhejiang People’s Republic of China

**Keywords:** Haploinsufficiency, NR3C1, Glucocorticoid resistance, Acute lymphoblastic leukemia, Mitochondrial apoptosis axis, Bcl-2 blockage

## Abstract

**Background:**

Relapse represents the leading cause of death in both child and adult patients with acute lymphoblastic leukemia (ALL). Development of chemo-resistance is ultimately responsible for treatment failure and relapse, therefore understanding the molecular basis underlying resistance is imperative for developing innovative treatment strategies. Glucocorticoids (GCs) such dexamethasone and prednisolone are the backbone of combination chemotherapy regimens for treating all lymphoid tumors. However, the biological mechanisms of primary GC resistance in ALL is not completely understood. We previously performed a longitudinal whole-exome sequencing analysis on diagnosis/relapse pairs from adult patients with ALL. Our data revealed that relapse-specific truncation mutations in the *NR3C1* gene, encoding the GC receptor, are frequently detected.

**Methods:**

In the current study, we used discovery-based strategies including RNA sequencing (RNA-seq) and CRISPR/Cas9, followed by confirmatory testing, in human ALL cell lines, bone marrow blast samples from ALL patients and xenograft models, to elucidate the mechanisms responsible for resistance.

**Results:**

Our results revealed a positive correlation between endogenous expression of *NR3C1* in ALL cells and sensitivity to GCs and clinical outcomes. We further confirmed that ectopic expression of *NR3C1* in ALL cells could reverse GC resistance, while deletion of *NR3C1* confers resistance to GCs in ALL cell lines and xenograft models. RNA-seq analysis revealed a remarkable abundance of gene signatures involved in pathways in cancer, DNA replication, mismatch repair, P53 signalling, cell cycle, and apoptosis regulated by *NR3C1.* Significantly increased expression of pro-apoptotic genes including *BCL2L11/Bim*, *BMF*, *BAD*, *BAX* and *BOK*, and decreased transcription of anti-apoptotic genes including *BCL2*, *BCL2L1* and *BAG2* were observed in GC-resistant ALL cells following ectopic expression of *NR3C1*. Finally, we explored that GC resistance in ALL cells with haploinsufficiency of *NR3C1* can be treated with Bcl-2 blockage.

**Conclusions:**

Our findings suggest that the status of *NR3C1* gene mutations and basal expression levels of *NR3C1* in ALL cells are associated with sensitivity to GCs and clinical treatment outcomes. Early intervention strategies by rational combination of Bcl-2 blockage may constitute a promising new treatment option to GC-resistant ALL and significantly improving the chances of treating poor prednisone responders.

## Background

Although steady improvements to chemotherapeutic treatments have helped cure 80% of childhood B-cell acute lymphoblastic leukemia (B-ALL) cases, chemotherapy has proven to be less effective for treating the majority of adult patients; the equivalent rate for adults is only 30–40% [1, 2], with relapse representing the leading cause of death in both children and adults. Development of chemo-resistance is ultimately responsible for treatment failure and relapse, so that a greater understanding of the molecular basis underlying this resistance is imperative for discovering innovative treatment strategies.

Relapse-specific/enriched genetic alterations are often apparent in clones that have gained an advantage under selective pressure from specific chemotherapeutics. We previously performed a longitudinal whole-exome sequencing analysis on diagnosis/relapse pairs from adult patients with B-ALL. Our data revealed frequent relapse-specific mutations in the *NR3C1* gene, encoding glucocorticoid receptor alpha, a nuclear receptor ligand-activated transcription factor [3]. Glucocorticoids (GCs) such dexamethasone and prednisolone are the backbone of combination chemotherapy regimens for treating all lymphoid tumours, which is further underscored by the strong association of primary GC resistance with poor prognosis in childhood ALL. More intriguingly, in our previous study, all relapse-specific mutations identified in the *NR3C1* gene were truncated mutations resulting in haploinsufficiency of the NR3C1 protein [3]. In the current study, we used discovery-based strategies including RNA sequencing (RNA-seq) and CRISPR/Cas9, followed by confirmatory testing approaches. We identified mitochondrial apoptotic signalling as a relevant mechanism responsible for chemo-resistance induced by the reduced expression of *NR3C1* in ALL cells, and showed that this can be pharmacologically treated by Bcl-2 blockage.

## Materials and methods

### Clinical samples, cell lines and reagents

Cryopreserved lymphoblast samples of bone marrow were collected at diagnosis or relapse from ALL patients from the Institute of Hematology of Zhejiang University (Hangzhou, China). Written informed consent was provided according to the Declaration of Helsinki. This study was approved by Clinical Research Ethics Committee of Sir Run Run Shaw Hospital, Zhejiang University School of Medicine (Approval No. 20180226-4). The authors have no conflicting financial interests.

Human ALL cell lines (Reh, Jurkat, CCRF-CEM, 6T-CEM, and NALM6) and HEK293T cells were purchased from the cell bank of the Chinese Academy of Science (Shanghai, China). Lymphoblastic leukemia cells were cultured in RPMI-1640 medium (Corning, Corning, NY, USA) supplemented with 10% fetal bovine serum (FBS; Gibco, Carlsbad, CA, USA). HEK293T cells were maintained in Dulbecco’s modified Eagle’s medium (DMEM; Corning) with 10% FBS. All cells were maintained at a density of < 5 × 10^5^ cells/ml and cultured at 37 °C in a humidified atmosphere of 95% air/5% CO_2_.

Monoclonal antibodies (mAbs) directed against NR3C1, Bcl-XL, Bim were purchased from Cell Signaling Technology (Danvers, MA, USA). Monoclonal antibodies recognising Bcl-2, Bad and Bax were obtained from Santa Cruz Biotechnology (Santa Cruz, CA, USA). Monoclonal antibody recognising Bak was purchased from Sigma-Aldrich (St. Louis, MO, USA). Bcl-2 inhibitor (ABT-263) was purchased from Selleck Chemicals (Houston, TX, USA). Dexamethasone was purchased from Sigma-Aldrich.

### Drug treatment, cell viability and cell apoptosis assay

ALL cell lines (1 × 10^5^ cells/well in 6-well plates) were treated with the respective drugs at concentrations of 0.1–5 µM or with dimethyl sulphoxide (DMSO) for 24–48 h. Cell viabilities were assessed using a Cell Counting Kit-8 (Dojindo, Kumamoto, Japan) and a microplate reader (Model 680; Bio-Rad, Hercules, CA, USA) according to the manufacturer’s instructions. To further analyse ALL cell apoptosis, ALL cell lines (3 × 10^5^ cells/well in 6-well plates) were treated with the respective drugs at the indicated concentrations and incubated for 48 h in RPMI-1640 medium supplemented with 10% FBS. For annexin-V apoptosis assays, cells were stained in 100 μl buffer (10 mM HEPES, 140 mM NaCl, 2.5 mM CaCl_2_) with 5 μl of APC annexin-V (cat. 550475; BD Biosciences, San Jose, CA, USA) and 5 µl 20 μg/ml 4′,6-diamidino-2-phenylindole (DAPI). Cells were stained for 15 min in the dark before the addition of 400 μl of the HEPES buffer. All analyses were performed by flow cytometry (Beckman Coulter, Inc., Miami, FL, USA).

### Plasmids and lentivirus infection

The C-terminal Myc-DDK-tagged pLenti plasmid carrying the full-length *Homo sapiens NR3C1* coding sequence (*NR3C1*, 2334 bp, NM_000176; hereafter called pLenti-C-Myc-DDK-NR3C1) was purchased from Biowestern Technologies (Hangzhou, China). Detailed methods for lentivirus infection have been described previously [4]. In brief, lentivirus was produced by cotransfection of packaging plasmids (PSPAX2 and PMD2.G) and lentivirus vectors into HEK293T cells using Attractene Transfection Reagent (Qiagen, Valencia, CA, USA) according to the manufacturer’s instructions. Supernatants containing lentivirus were harvested at 72 h after transfection, filtered through a 4.5 μm filter, and purified using 40% polyethylene glycol (PEG) 8000 (10% final concentration; Sigma-Aldrich). Lentivirus solution were added to the cells after being diluted in 1 ml complete medium containing 8 mg/ml polybrene (Sigma-Aldrich), and incubated for 12 h at 37 °C, followed by incubation in 1 ml of fresh complete medium. Positive clones were selected with 10 μg/ml blasticidin (Invitrogen, Waltham, MA, USA) at day 5 after infection. Transfection efficiencies were > 80%.

### CRISPR guide RNA vector construction and clone isolation

Two CRISPR single-guide RNAs (sgRNAs) sgRNA1 (5′-CACCGGCTGAACT CTTGGGGTTCTC-3′) and sgRNA2 (5′-CACCGCTCTCATTCGTCTCTTTACC-3′) targeting the second exon of the *NR3C1* gene were designed according to the recommendations on the Zhang laboratory website (http://crispr.genomeengineering.org) and cloned into the pSpCas9 (BB)-2A-GFP vector (PX458; Addgene). *NR3C1* sgRNA vectors were electroporated into ALL cells using the NEON transfection system (Invitrogen). After 48–72 h, cells were sorted for expression of green fluorescent protein (GFP) using a BD FACSAria cell sorter (BD Biosciences). After confirmation of gene knock-in via PCR amplification (forward primer 5′-CTCAGTAAGCAATGCGCAGC-3′, reverse primer 5′-ACACTGATCTTACCTT GAATAG CC-3′) and DNA sequencing, the rest of the sorted cells were plated on 96-well plates for single-clone isolation. Positive single clones were selected and confirmed by PCR amplification and DNA sequencing.

### Transcriptome sequencing and gene expression level measurements

Total RNA was extracted from cell samples using TRIzol Reagent (Life Technologies, Carlsbad, CA, US) following the manufacturer’s instructions. For sequencing library preparation, the VAHTS mRNA-seq v2 Library Prep Kit for Illumina (NR601; Vazyme, Nanjing, China) was used according to the manufacturer’s protocol. Briefly, total mRNA was purified with poly-T oligo-attached magnetic beads. Fragmentation was performed using divalent cations in Vazyme Frag/Prime Buffer with elevated temperature. First-strand cDNA was reverse transcribed using random primers. Second-strand cDNA synthesis was subsequently performed using dNTPs, DNA polymerase I, RNase H and buffer. Following end repair with the addition of a single ‘A’ base at the 3′ end of each strand and adaptor ligation with special sequencing adapters (N803; Vazyme), products were purified and size-selected with VAHTS DNA Clean Beads (N411; Vazyme) to obtain appropriate sizes for sequencing.

RNA-seq was performed on an Illumina Hiseq X Ten platform (Illumina, San Diego, CA, USA) and a 150-bp paired-end module. The reference genome index was built using Hisat2-build, and paired-end clean reads were then processed and aligned to the reference genome using Hisat2 (v.2.0.5) [5, 6]. Mapped reads for each sample were assembled into transcriptome data using Stringtie (v.1.3.3) with a reference-based approach [5, 7]. This method employed spliced reads to determine exon connectivity.

Fragments per kilobase of exon per million fragments mapped (FPKM) values were calculated based on the fragments’ length and read counts mapped to each fragment. FPKMs for coding genes in each sample were calculated using Cuffdiff (v.1.3.0) [8]. Transcriptome FPKMs were computed by summing the FPKMs of transcripts in each gene group. Significantly different expression in digital transcript or gene expression datasets were determined by a model based on a negative binomial distribution with Cuffdiff (v.2.2.1). Only comparisons with *q*-values < 0.05 and absolute log2 (fold change) values ≥ 1 were considered significantly differentially expressed.

### Reverse-transcription quantitative-PCR (RT-qPCR) and western blotting validation of transcriptome-seq results

RT-qPCR and western blotting analyses were carried out as previously described [4] with *NR3C1* primers 5′-ATAGCTCTGTTCCAGACTCAACT-3′ (forward) and 5′-TCCTGAAACCTGGTATTGCCT-3′ (reverse). *GAPDH* primers were 5′-ACAACTTTGGTATCGTGGAAGG-3′ (forward) and 5′-GCCATCACGCCACAGTTTC-3′ (reverse).

### Xenograft model studies

All animal studies were carried out following the approval of animal ethical approval from Ethics Committee of Sir Run Run Shaw Hospital, Zhejiang University School of Medicine (Approval No. 11954). NOD-Prkdc^scid^/IL2rg^tm1^/Bcgen (B-NSG/B-NDG) mice were purchased from BIOCYTOGEN (Jiangsu, China). Male mice at 8-weeks old were inoculated by tail vein injection with 1 × 10^6^ wild-type NALM6 ALL cells, NALM6 ALL cells harbouring knockdown of the *NR3C1* gene, Reh ALL cells overexpressing the *NR3C1* gene, or Reh ALL (control) cells, respectively. Leukemia engraftment was monitored by weekly enumeration of the proportion of human CD45^+^ cells (for Reh cell cohorts) or human CD19^+^ cells (for NALM6 cell cohorts) in peripheral blood (%huCD45^+^ or  %huCD19^+^). When median %huCD45^+^ or %huCD19^+^ values reached 1%, treatment was initiated; dexamethasone was administered at 15 mg/kg for 5 days via intraperitoneal injection. Leukemia progression was monitored following completion of treatment of drug. An event was defined that animals exhibited signs of morbidity related to high leukemic infiltration of bone marrow and spleen. To allow comparisons between dexamethasone-susceptible and -resistant groups, overall survival (OS) was calculated for each mouse and subjected to Kaplan–Meier analysis.

### Statistical analysis

Data are expressed as the mean ± standard deviation (SD), and each group was consisted of three biological replicates. Different groups were compared using Fisher’s exact tests or independent-samples t-tests. SPSS software version 16.0 (IBM, Armonk, NY, USA) was used to perform statistical analyses. All probability values were generated from two-sided tests. A *p*-value < 0.05 was considered to be statistically significant.

## Results

### Association between endogenous expression of *NR3C1* in ALL cells and both sensitivity to glucocorticoids and clinical treatment outcomes

**Fig. 1:**
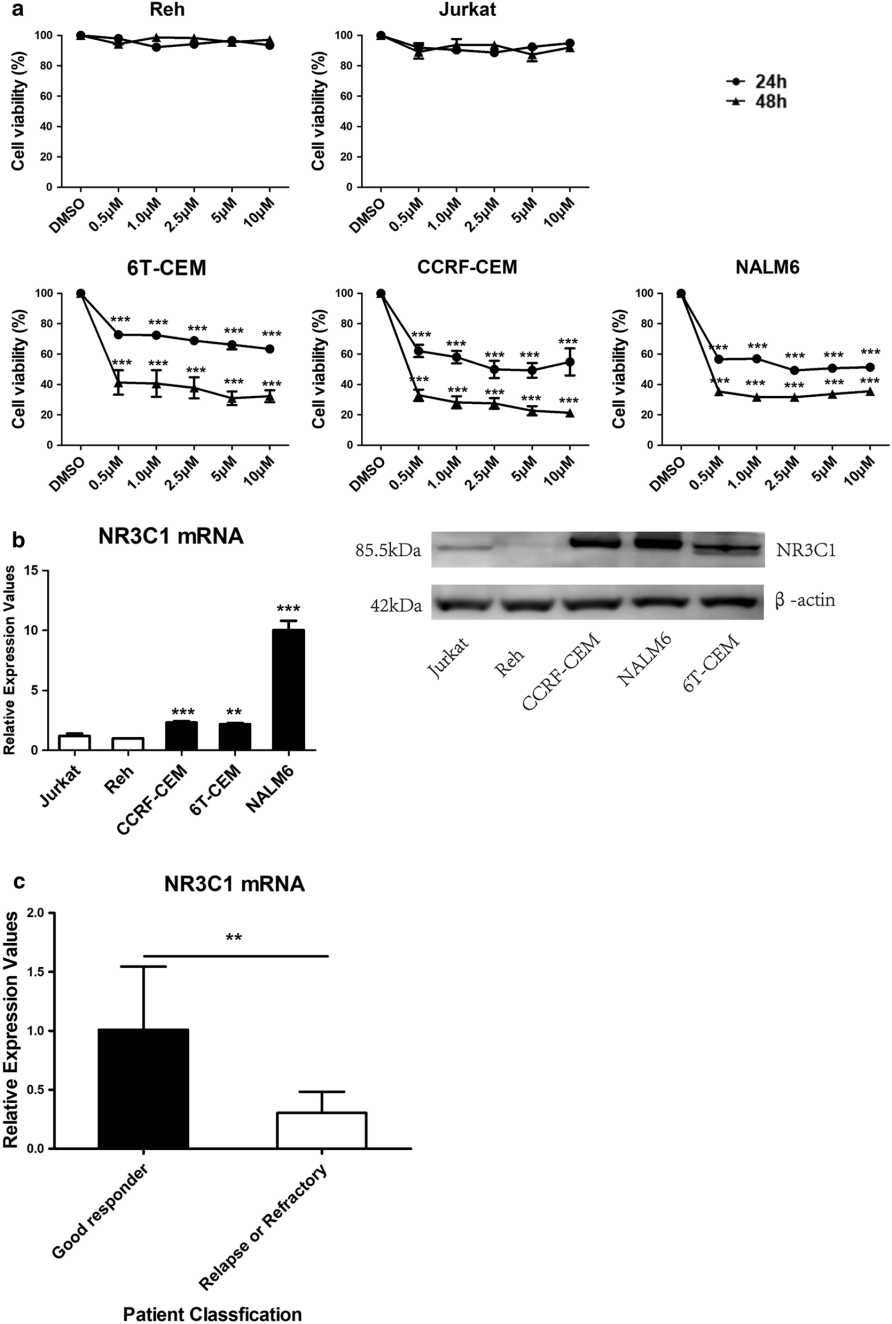
Association between endogenous expression of *NR3C1* in ALL cells and sensitivity to glucocorticoids and clinical treatment outcomes. **a** Effects of dexamethasone on human ALL cell proliferation. Shown are summarized results of cell viability assays in ALL cell lines treated with different concentrations (0.1, 0.5, 1.0, 2.5 or 5 μmol/l) of dexamethasone after 24–48 h. Statistical analyses compared cells treated with DMSO and those treated with the indicated drugs using *t*-tests (unpaired, two-sided). The data are presented as means of 3 replications. **b** RT-qPCR (left) and western blotting (right) analysis of endogenous *NR3C1* expression in ALL cell lines. **c** Comparison of *NR3C1* mRNA expression in bone marrow blast samples from patients of good responders to prednisolone (n = 19) and patients with refractory or relapsed ALL (n = 30). Error bars represent standard deviation (SD). *p < 0.05, **p < 0.01, ***p < 0.001

We first investigated the association between endogenous expression of *NR3C1* and sensitivity to dexamethasone using in vitro-cultured human ALL cell lines. Reh, Jurkat, CCRF-CEM, NALM6 and 6T-CEM cells in the log growth phase were collected and treated with different concentrations (0.1, 0.5, 1.0, 2.5 or 5 μmol/l) of dexamethasone. After 24–48 h treatment, cell proliferation of CCRF-CEM, 6T-CEM and NALM6 cells was significantly inhibited by dexamethasone in both a time- and dose-dependent manner. By contrast, cell proliferation of Reh and Jurkat cells was not reduced (Fig. 1a). RT-qPCR and western blotting analysis showed that endogenous *NR3C1* expression in dexamethasone-sensitive CCRF-CEM, 6T-CEM and NALM6 cell lines was significantly higher than in that in dexamethasone-resistant Reh and Jurkat cell lines (Fig. 1b).

We then performed expression analysis of *NR3C1* in bone marrow (BM) blast samples obtained from 49 adult ALL patients at diagnosis or relapse. The endpoint of the last follow-up for all the surviving patients was December 31, 2018. Patients were divided into two cohorts; 19 patients were positive responders to prednisolone (good-responder cohort) and achieved persistent complete remission after a median follow-up time of 6.8 months (with a range of 3.3–14 months); 30 patients were refractory or relapsed ALL (R/R ALL cohort), of which nine patients were primary refractory ALL and responded poorly to induction chemotherapy, and 21 patients experienced relapse during consolidation treatment (n = 16) or following allogeneic hematopoietic stem cell transplantation (n = 5). The mean mRNA expression of *NR3C1* in ALL cells of patients in the good-responder cohort was 3.3-fold higher than in R/R leukemia cells (*p* < 0.01; Fig. 1c). Our results suggest that the sensitivity of ALL cells to GCs was positively associated with endogenous *NR3C1* expression.

### Ectopic expression of *NR3C1* in glucocorticoid-resistant ALL cells can reverse resistance in vitro and in vivo

**Fig. 2:**
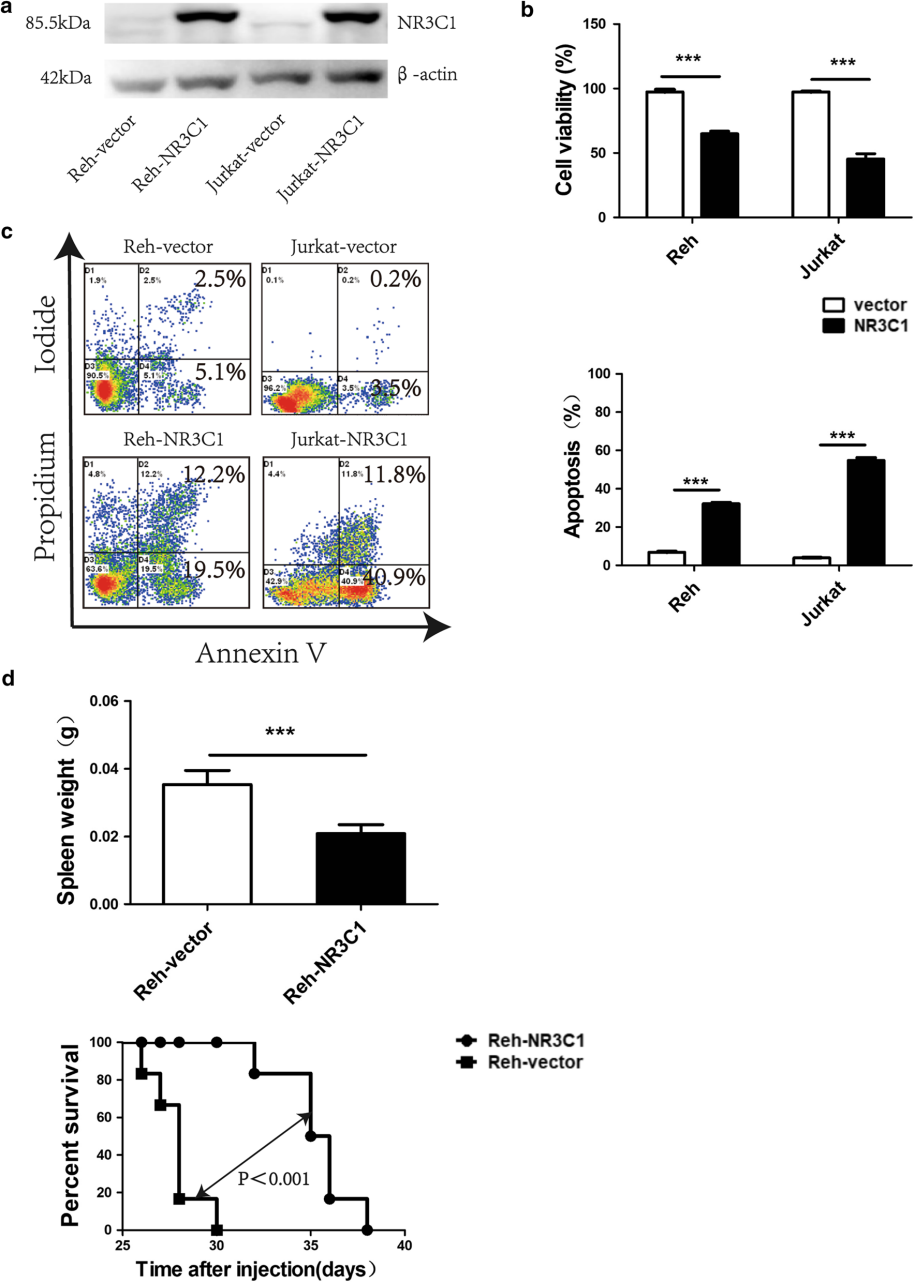
Effects of ectopic expression of *NR3C1* in glucocorticoid-resistant ALL cells. **a** Reh and Jurkat ALL cells were transfected with pLenti-C-Myc-DDK-vector (control), pLenti-C-Myc-DDK-NR3C1. After 5 days, cells were then harvested and analyzed by western blotting with anti-NR3C1 or β-actin antibody. **b** Results of cell viability assays in transfected cells treated with 1 μmol/l dexamethasone for 48 h. **c** Results of apoptosis assays in transfected cells treated with 1 μmol/l dexamethasone for 48 h. **d** Results of leukemic infiltration of spleen, and overall survival in immunodeficient B-NSG mice injected with Reh cells overexpressing the *NR3C1* gene or Reh cells transfected with the empty vector. *p < 0.05, **p < 0.01, ***p < 0.001

We hypothesised that overexpression of *NR3C1* might induce dexamethasone sensitivity in ALL cells. To address this question, we chose the two dexamethasone-resistant Reh and Jurkat ALL cell lines and transfected each with the wild-type *NR3C1* expression vector pLenti-C-Myc-DDK-NR3C1. As shown in Fig. 2a, the concentration of NR3C1 protein was successfully increased in both Reh-NR3C1 and Jurkat-NR3C1 cells according to western blotting analysis. We then examined the rates of cell proliferation and apoptosis in transfected cells after treatment with 1 μmol/l dexamethasone for 48 h. As shown in Fig. 2b, c, compared with cells transfected with empty vector as a control, Reh-NR3C1 displayed a lower cell proliferation rate (64 ± 3% vs. 96 ± 2%; *p* < 0.001) and a higher rate of cell apoptosis (33.6 ± 0.3% vs. 6.5 ± 0.1%; *p* < 0.001). Jurkat cells showed the same effect, with rates of cell proliferation and apoptosis of 45 ± 7.2% and 57.4 ± 0.8% in the transfected cohorts vs. 97 ± 0.6% and 4.23 ± 0.3% in the control cohorts, respectively (*p* < 0.001).

Next, we confirmed the association between expression of *NR3C1* and sensitivity to dexamethasone in vivo in a xenograft ALL model. Reh cells overexpressing the *NR3C1* gene or Reh cells transfected with the empty vector were intravenously injected into immunodeficient B-NSG mice. Animals harbouring Reh-NR3C1 showed significantly decreased spleen weight, and experienced longer overall survival after treatment with dexamethasone compared with empty vector controls (Fig. 2d).

### Deletion of *NR3C1* reduces sensitivity to glucocorticoids in vitro and in vivo

**Fig. 3:**
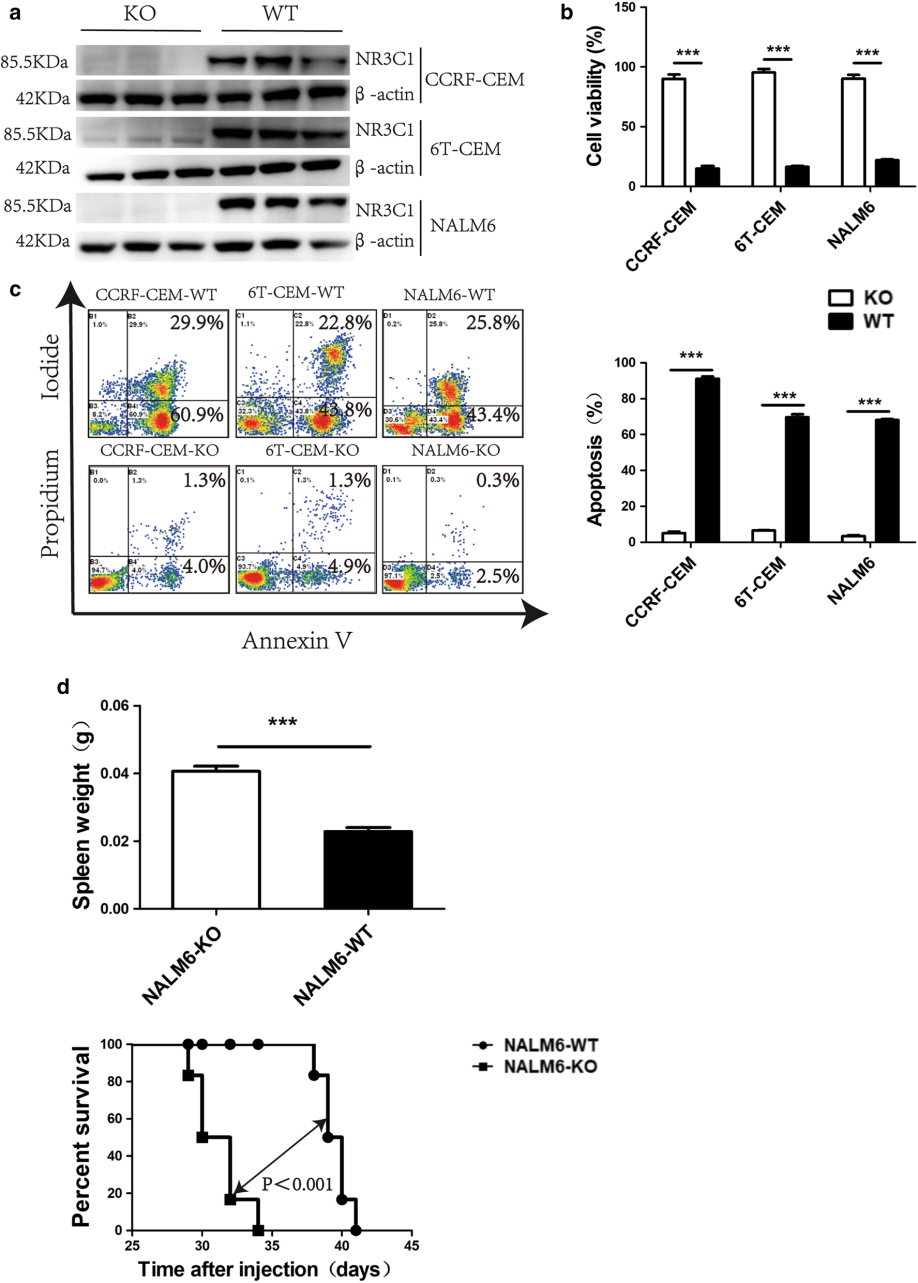
Effects of deletion of *NR3C1* in glucocorticoid-sensitive ALL cells. **a** CCRF-CEM, 6T-CEM and NALM6 ALL cells were knocked down the *NR3C1* gene by CRISPR guide RNA vector transfection. Positive single clones (CCRF-CEM-KO, 6T-CEM-KO and NALM6-KO) were chosen and confirmed by western blotting analysis with anti-NR3C1 or β-actin antibody. **b** Results of cell viability assays in wild type ALL cells and *NR3C1* knockdown cells treated with 1 μmol/l dexamethasone for 48 h. **c** Results of apoptosis assays in wild type ALL cells and *NR3C1* knockdown cells treated with 1 μmol/l dexamethasone for 48 h. **d** Results of leukemic infiltration of spleen, and overall survival in immunodeficient B-NSG mice injected with NALM6 cells with *NR3C1* gene knockdown or wild type NALM6 cells. *p < 0.05, **p < 0.01, ***p < 0.001

We chose three dexamethasone-sensitive CCRF-CEM, 6T-CEM and NALM6 ALL cell lines and knocked down the *NR3C1* gene by CRISPR guide RNA vector transfection. Positive single clones were chosen and confirmed by DNA sequencing and western blotting analysis (Fig. 3a, Additional file 1: Figure S1). After treatment with 1 μmol/l dexamethasone for 48 h, compared with the negative control group, CCRF-CEM cells with *NR3C1* knockdown (CCRF-CEM-KO) displayed a significantly higher rate of cell proliferation (93 ± 12% vs. 15 ± 4%; *p* < 0.001) and reduced cell apoptosis (5.1 ± 1.7% vs. 79.4 ± 15.4%; *p* < 0.001). The same tendency was also observed when disabling the *NR3C1* gene in 6T-CEM cells and NALM6 cells (Fig. 3b, c).

We also found that immunodeficient B-NSG mice harbouring NALM6 cells with *NR3C1* knockdown (NALM6-KO) showed significantly increased spleen weight, and experienced poorer overall survival after treatment with dexamethasone, compared with the empty vector controls (Fig. 3d).

### *NR3C1* regulates glucocorticoid sensitivity via disruption of the mitochondrial apoptosis axis in ALL cells

**Fig. 4:**
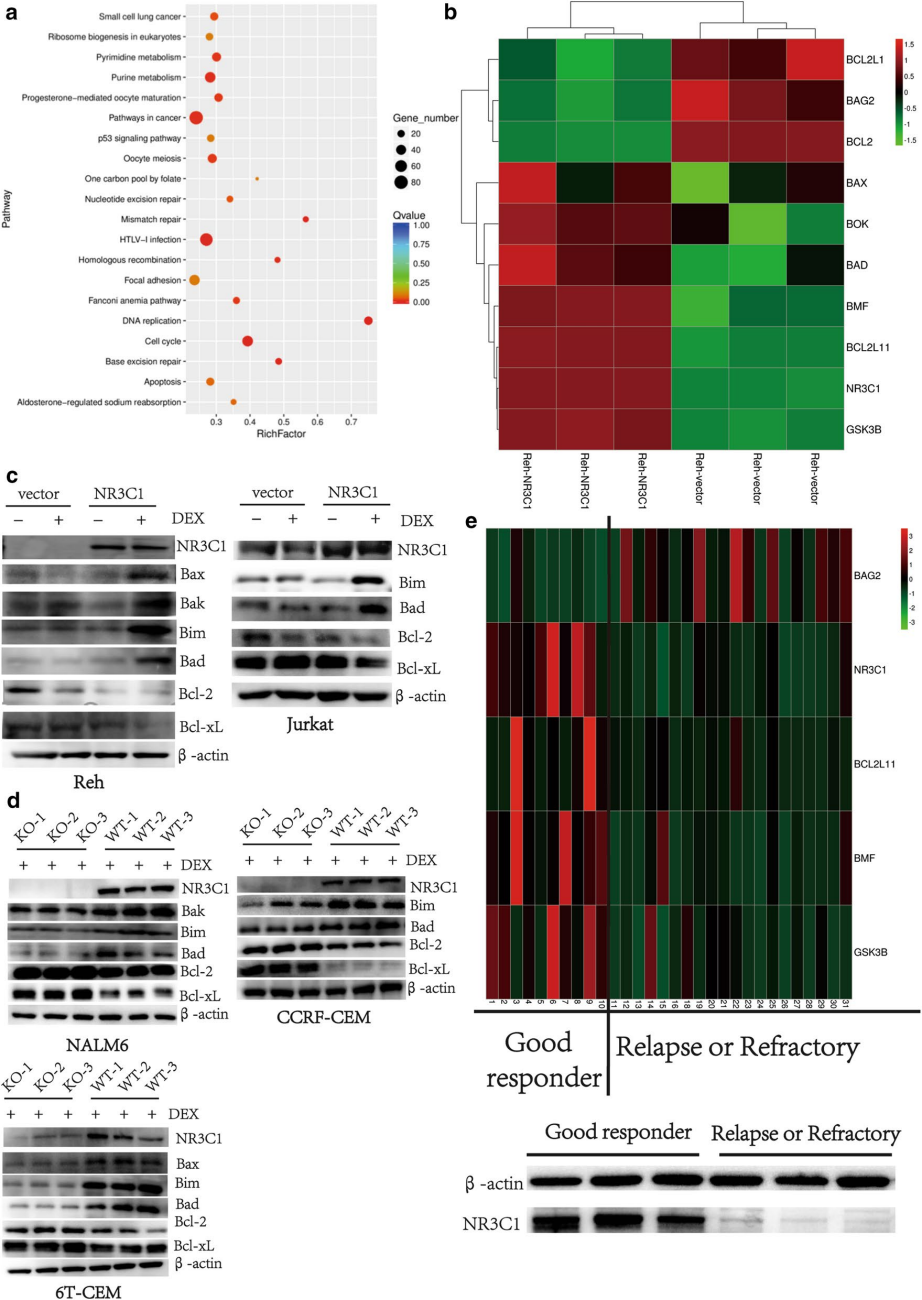
*NR3C1* disrupts the mitochondrial apoptosis axis in ALL cells. **a** Transcriptome sequencing of ex vivo-cultured wild-type Reh cells (GC-resistant) and Reh cells with ectopic expression of *NR3C1* (GC-sensitive) following exposure to dexamethasone. Pathway analysis of genes regulated by *NR3C1* in the indicated ALL cell line. **b** Significantly increased expression of pro-apoptotic genes and decreased transcription of anti-apoptotic genes were observed in Reh cells with ectopic expression of *NR3C1*. **c** Western blotting analysis of expression of the pro-apoptotic proteins and anti-apoptotic proteins in GC-resistant Reh and Jurkat ALL cells ectopically expressing *NR3C1*. **d** Western blotting analysis of expression of the pro-apoptotic proteins and anti-apoptotic proteins in GC-sensitive CCRF-CEM, 6T-CEM and NALM6 cells with *NR3C1* gene knockdown. **e** Comparison of mRNA expression of the pro-apoptotic genes and anti-apoptotic genes in bone marrow blast samples from patients of good responders to prednisolone (n = 10) and patients with refractory or relapsed ALL (n = 20). In the bottom, western blotting analysis of the concentration of NR3C1 protein in ALL cells from 3 patients in the good-responder cohort and from 3 R/R ALL patients

To explore candidate genes regulated by *NR3C1* following GC treatment, we performed transcriptome sequencing of ex vivo-cultured wild-type Reh cells (GC-resistant) and Reh cells with ectopic expression of *NR3C1* (GC-sensitive) following exposure to dexamethasone. Data analysis revealed 3935 genes that were significantly (log2Ratio| ≥ 1, *q *< 0.05) differentially expressed, relative to wild-type Reh controls, in Reh cells with ectopic expression of *NR3C1*. Pathway analysis revealed a remarkable abundance of gene signatures involved in pathways in cancer, DNA replication, mismatch repair, P53 signalling, cell cycle and apoptosis regulated by *NR3C1* (Fig. 4a). Further analysis identified the mitochondrial apoptosis axis as the process most altered at the gene expression level. Significantly increased expression of pro-apoptotic genes, including *BCL2L11/Bim*, *BMF*, *BAD*, *BAX* and *BOK*, and decreased transcription of anti-apoptotic genes, including *BCL2*, *BCL2L1* and *BAG2,* were observed in Reh cells ectopically expressing *NR3C1* (Fig. 4b). Western blotting analysis confirmed that overexpression of *NR3C1* in GC-resistant Reh and Jurkat ALL cell lines could induce expression of the pro-apoptotic proteins Bim, Bad, Bax and Bak, and decrease expression of the anti-apoptotic proteins Bcl-2 and Bcl-xl (Fig. 4c). When the *NR3C1* gene was disabled in GC-sensitive CCRF-CEM, 6T-CEM and NALM6 cell lines, we also identified decreased expression of pro-apoptotic proteins and increased expression of anti-apoptotic proteins (Fig. 4d).

We also analysed expression of *NR3C1*-regulated genes in specimens from the aforementioned 49 ALL patients. The concentration of NR3C1 protein in ALL cells of patients in the good-responder cohort was higher than in R/R leukemia cells according to western blotting analysis (Fig. 4e bottom). We further confirmed that ALL cells from patients in the good-responder cohort displayed significantly higher expression of pro-apoptotic genes and significantly lower expression of anti-apoptotic genes than did ALL cells from the R/R ALL cohort (Fig. 4e).

### Glucocorticoid resistance in ALL cells with *NR3C1* haploinsufficiency is sensitive to Bcl-2 blockage

**Fig. 5:**
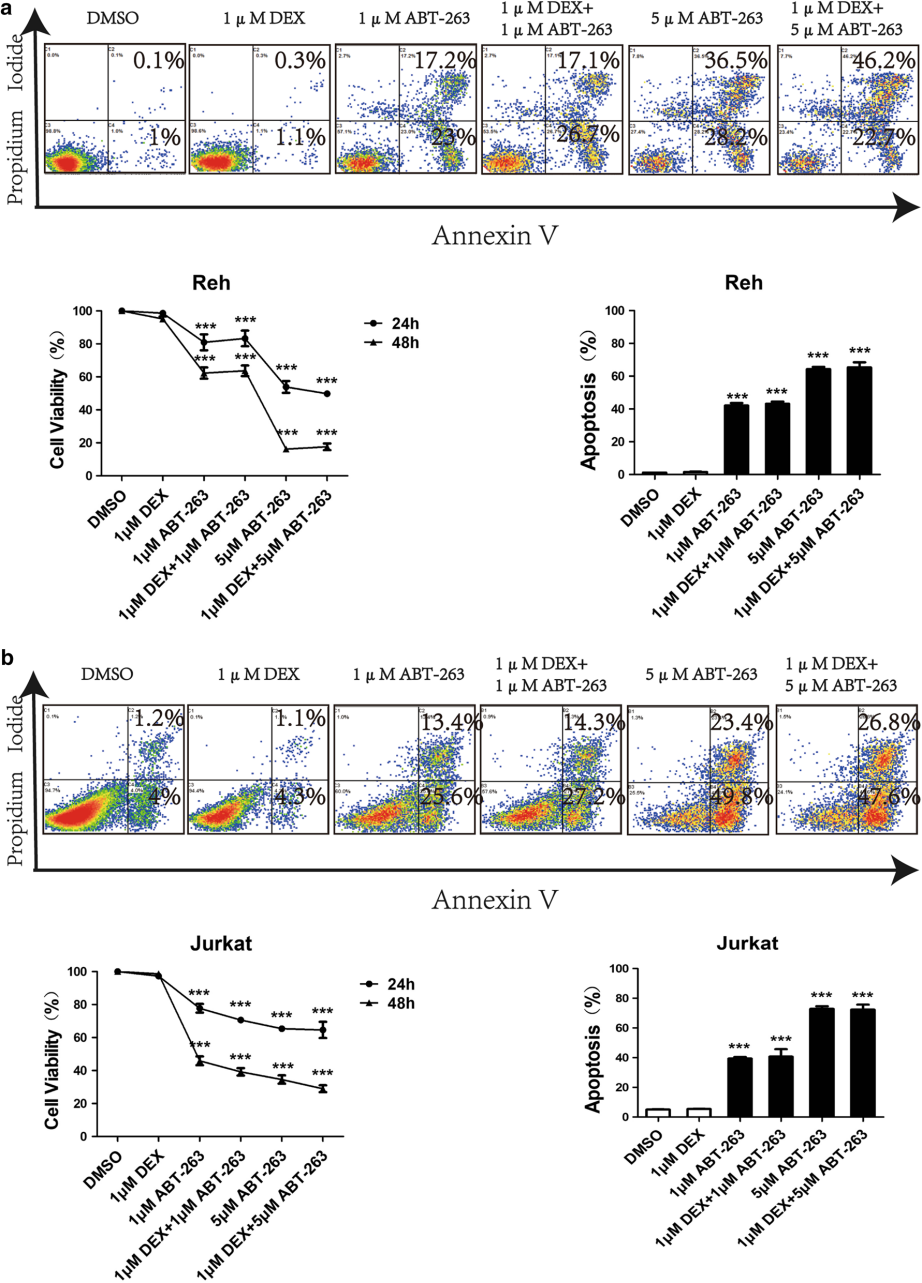
Effects of Bcl-2 blockage in glucocorticoid-resistant ALL cells. **a** Results of cell viability assays in Reh and Jurkat ALL cell lines treated with different concentrations (1 or 5 μmol/l) of the Bcl-2 inhibitor ABT-263, dexamethasone, or dexamethasone plus ABT-263 for 24–48 h. **b** Results of apoptosis assays in Reh and Jurkat ALL cell lines treated with different concentrations (1 or 5 μmol/l) of the Bcl-2 inhibitor ABT-263, dexamethasone, or dexamethasone plus ABT-263 for 48 h. Statistical analyses compared cells treated with dexamethasone and those treated with ABT-263, dexamethasone plus ABT-263 using *t*-tests (unpaired, two-sided). The data are presented as means of 3 replications. *p < 0.05, **p < 0.01, ***p < 0.001

We found that Bcl-2 is a major negative regulator of *NR3C1* activity and thereby drives GC sensitivity in ALL cells. Thus, we tested the therapeutic role of Bcl-2 inhibition in the treatment of GC-resistant leukaemia cells. We treated GC-resistant Reh and Jurkat ALL cell lines with different concentrations (1 or 5 μmol/l) of the Bcl-2 inhibitor ABT-263, dexamethasone, or dexamethasone plus ABT-263. In these experiments, compared with cells treated with 1 μmol/l dexamethasone, ABT-263 in Reh or Jurkat lymphoblasts effectively induced apoptosis and inhibition of cell proliferation in vitro. After treatment with ABT-263 for 48 h, Reh cells displayed a higher rate of cell apoptosis compared with cells treated with dexamethasone (for 1 μmol/l ABT-263, 42.6 ± 1.59% vs. 1.4 ± 0.25%; *p* < 0.001; for 5 μmol/l ABT-263, 64.7 ± 1.35% vs. 1.4 ± 0.25%; *p* < 0.001). The same tendency was also observed in Jurkat cells (for 1 μmol/l ABT-263, 39 ± 1.09% vs. 5.4 ± 0.25%; *p* < 0.001; for 5 μmol/l ABT-263, 73.2 ± 1.72% vs. 5.4 ± 0.25%; *p* < 0.001). However, no additive effect was observed in dexamethasone in combination with ABT-263 (Fig. 5a, b).

## Discussion

Synthetic glucocorticoids (GCs) play a fundamental role in the treatment of all lymphoid tumours due to their capacity to induce apoptosis in lymphoid progenitor cells [9, 10]. Resistance to GCs is a major clinical problem in the treatment of ALL. Numerous mechanisms have been proposed to explain the lack of effective GC-induced apoptosis in poor prednisone responders [11]. However, the molecular basis of primary GC resistance in ALL is not completely understood.

The GC-induced apoptotic response is mediated through the GC receptor (GCR), a member of the nuclear receptor family of ligand-dependent transcription factors. Although there is only one known gene encoding this receptor, *NR3C1* (located on chromosome 5q31.3), several receptor isoforms result from alternative splicing [12, 13]. Whether mutations in the GCR gene contribute to GC resistance in patients is controversial. Previous studies suggested that GCR mutations are rarely seen in samples from patients at relapse. In an older study, using nine T-ALL cell lines, single-nucleotide polymorphism analysis of the *NR3C1* coding region identified no mutations known to be associated with GC resistance [14]. In another study [15], mutational screening of all coding exons of the *NR3C1* gene was performed alongside loss of heterozygosity (LOH) analyses in a cohort of lymphoblast samples from 50 relapsed ALL patients. The results showed that somatic mutations and LOH of the GCR rarely contribute to relapsed disease in children with ALL [15]. However, loss of the *NR3C1* gene has recently been reported in 10% of childhood *ETV6/RUNX1*-positive ALL, and reflects a poor response to induction treatment, which possibly accounts for the adverse prognosis of some *ETV6/RUNX1*-positive relapses [16, 17]. Furthermore, *NR3C1* gene deletions were found at diagnosis (conserved at relapse), and many prevailed at relapse [17, 18]. We also previously identified truncated mutations of the *NR3C1* gene exclusively at relapse in normal karyotype adult ALL patients [3]. However, there is conflicting evidence as to whether GC sensitivity is associated with basal GCR levels [11]. In two previous studies [19, 20], GCR expression level and GC-induced regulation of both pro-apoptotic and anti-apoptotic pathway components were found to play a major role in the sensitivity of patient-derived ALL cells and ALL cell lines to GC. More recently, decreased GCR protein levels were found to be a major determinant of de novo corticosteroid resistance in B-ALL [21] and multiple myeloma [22]. Moreover, corticoresistance could be driven by haploinsufficiency of *NR3C1* expression in blastic plasmacytoid dendritic cell neoplasms [23]. In line with these reports, our clinical and functional analyses in vitro and in vivo showed that low *NR3C1/GCR* expression is linked to poor prognosis and tumour progression. Conversely, forced expression of *NR3C1* overcomes drug resistance in leukemic cells.

GCs regulate the expression of target genes by binding to the GCR, triggering its translocation into the nucleus and association with a GC-response element, thereby increasing or decreasing gene transcription. We delineated the ability of GCs to influence global gene expression in the presence or absence of *NR3C1*-enforced expression in a well-defined corticoresistant ALL in vitro model. Our study revealed that *NR3C1* regulated a remarkable array of gene signatures involved in pathways in cancer, DNA replication, mismatch repair, P53 signalling, cell cycle and apoptosis. A critical role for disequilibrium of pro- and anti-apoptotic proteins in GC-induced apoptosis of malignant lymphocytes has been well established [24–26]. However, a pro-apoptotic BH3-only member of the BCL-2 family, BCL-2-interacting mediator of cell death (BIM), is the only BCL-2 family member consistently shown in microarray analysis to be up-regulated by GCs in lymphoid cells [27, 28]. Our transcriptome sequencing and western blotting analysis results provide novel links between the GCR and the expression of pro/anti-apoptotic proteins involving BCL2L11/Bim, BMF, BAD, BAX, BOK, BCL2, BCL2L1 and BAG2, which are likely to be important in the mechanisms of GC resistance in lymphoid malignancies. Finally, we have proposed for the first time that the BH3-mimetic drug ABT-263, which was specifically designed to inhibit pro-survival BCL2 family proteins, and has shown significant efficacy in preclinical xenograft models of ALL [29–31], may play a potential role in the treatment of GC-resistant ALL cells.

## Conclusions

Our findings suggest that the status of *NR3C1* gene mutations and basal levels of *NR3C1* in ALL cells are associated with sensitivity to GCs and clinical treatment outcomes. Early intervention strategies by rational combination of Bcl-2 blockage may constitute a promising new treatment option to GC-resistant ALL and significantly improving the chances of treating poor prednisone responders. Randomised prospective trials are necessary to identify the optimal dose and treatment schedule.

## Supplementary information

**Additional file 1: Figure S1.** CCRF-CEM, 6T-CEM and NALM6 ALL cells were knocked down the *NR3C1* gene by CRISPR/Cas9 gene-editing methods. Positive single clones were chosen and confirmed by DNA sequencing.

## Data Availability

Data sharing not applicable to this article as no datasets were generated or analyzed during the current study. The datasets used and/or analyzed during the current study are available from the corresponding author on reasonable request.

## Abbreviations

ALL: acute lymphoblastic leukemia
GCs: glucocorticoids
RNA-seq: RNA sequencing
mAbs: monoclonal antibodies
DMSO: dimethyl sulphoxide
FPKM: fragments per kilobase of exon per million fragments mapped
RT-qPCR: reverse-transcription quantitative-PCR
BM: bone marrow
GCR: glucocorticoid receptor
