# Supplementary figures and images for: Haploinsufficiency of NR3C1 drives glucocorticoid resistance in adult acute lymphoblastic leukemia cells by down-regulating the mitochondrial apoptosis axis, and is sensitive to Bcl-2 blockage
Source: Cancer Cell Int. 2019 Aug 23;19:218. doi: 10.1186/s12935-019-0940-9 (PMC6708234; doi:10.1186/s12935-019-0940-9)

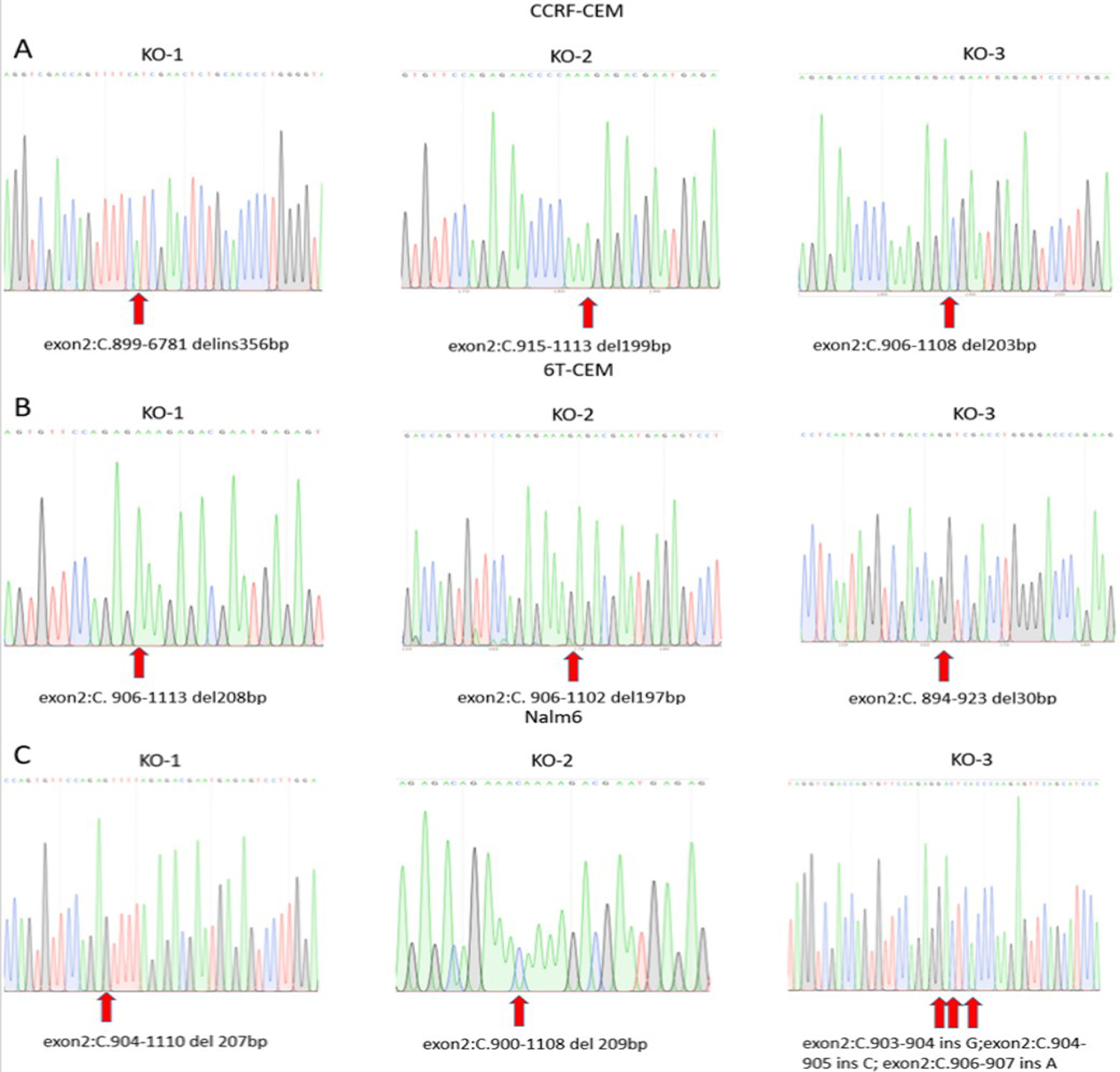

Supplement: Supplementary file 1 — Additional file 1: Figure S1. CCRF-CEM, 6T-CEM and NALM6 ALL cells were knocked down the NR3C1 gene by CRISPR/Cas9 gene-editing methods. Positive single clones were chosen and confirmed by DNA sequencing. [file 12935_2019_940_MOESM1_ESM.jpg]
